# Adenosine A_2A_ receptor antagonists act at the hyperoxic phase to confer protection against retinopathy

**DOI:** 10.1186/s10020-018-0038-1

**Published:** 2018-07-31

**Authors:** Rong Zhou, Shuya Zhang, Xuejiao Gu, Yuanyuan Ge, Dingjuan Zhong, Yuling Zhou, Lingyun Tang, Xiao-Ling Liu, Jiang-Fan Chen

**Affiliations:** 1 Institute of Molecular Medicine, School of Optometry and Ophthalmology and Eye Hospital, Wenzhou Medical University, 270 Xueyuan Road, Wenzhou, 325027 Zhejiang China; 2 State Key Laboratory Cultivation Base and Key Laboratory of Vision Science, Ministry of Health, China and Zhejiang Provincial Key Laboratory of Ophthalmology and Optometry, Wenzhou, Zhejiang China

**Keywords:** Retinopathy of prematurity, Oxygen-induced retinopathy, Adenosine A_2A_ receptor, KW6002

## Abstract

**Background:**

Retinopathy of prematurity (ROP) remains a major cause of childhood blindness and current laser photocoagulation and anti-VEGF antibody treatments are associated with reduced peripheral vision and possible delayed development of retinal vasculatures and neurons. In this study, we advanced the translational potential of adenosine A_2A_ receptor (A_2A_R) antagonists as a novel therapeutic strategy for selectively controlling pathological retinal neovascularization in oxygen-induced retinopathy (OIR) model of ROP.

**Methods:**

Developing C57BL/6 mice were exposed to 75% oxygen from postnatal (P) day 7 to P12 and to room air from P12 to P17 and treated with KW6002 or vehicle at different postnatal developmental stages. Retinal vascularization was examined by whole-mount fluorescence and cross-sectional hematoxylin-eosin staining. Cellular proliferation, astrocyte and microglial activation, and tip cell function were investigated by isolectin staining and immunohistochemistry. Apoptosis was analyzed by TUNEL assay. The effects of oxygen exposure and KW6002 treatment were analyzed by two-way ANOVA or Kruskal-Wallis test or independent Student’s t-test or Mann-Whitney U test.

**Results:**

The A_2A_R antagonist KW6002 (P7-P17) did not affect normal postnatal development of retinal vasculature, but selectively reduced avascular areas and neovascularization, with the reduced cellular apoptosis and proliferation, and enhanced astrocyte and tip cell functions in OIR. Importantly, contrary to our prediction that A_2A_R antagonists were most effective at the hypoxic phase with aberrantly increased adenosine-A_2A_R signaling, we discovered that the A_2A_R antagonist KW6002 mainly acted at the hyperoxic phase to confer protection against OIR as KW6002 treatment at P7-P12 (but not P12-P17) conferred protection against OIR; this protection was observed as early as P9 with reduced avascular areas and reduced cellular apoptosis and reversal of eNOS mRNA down-regulation in retina of OIR.

**Conclusions:**

As ROP being a biphasic disease, our identification of the hyperoxic phase as the effective window, together with selective and robust protection against pathological (but not physiological) angiogenesis*,* elevates A_2A_R antagonists as a novel therapeutic strategy for ROP treatment.

## Background

Retinopathy of prematurity (ROP) remains a major cause of childhood blindness in many parts of the world (Chen et al. 2008; Gilbert 2008; Dhaliwal et al. 2009; Husain et al. 2013; Smith et al. 2013; Hellstrom et al. 2013; Hartnett 2015). As this disease of premature infants is believed to be largely driven by hypoxia-induced factor-1α (HIF-1α) signaling pathway and vascular endothelial growth factor (VEGF) levels in retina (Penn et al. 2008; Cavallaro et al. 2014; Campochiaro 2015), with characteristic hypoxia-induced pathological angiogenesis (Cavallaro et al. 2014; Fleck and McIntosh 2008; Hartnett and Penn 2012), current study of therapeutic development for ROP has largely focused on anti-VEGF therapy. Anti-VEGF antibody has been shown successful in a clinical trial to reduce the recurrence rate in stage III of ROP infants (Mintz-Hittner et al. 2011). However, intraocular injections of anti-VEGF antibody are invasive and repeated injection is associated with the risk of endophthalmitis. Importantly, as VEGF being an important cellular survival factor, the anti-VEGF treatment has been associated with serious concerns about the unintended effects of anti-VEGF agents on delayed development of retinal vasculatures and neurons, and on brain development of preterm infants (Nishijima et al. 2007; Saint-Geniez et al. 2008). Thus, alternative and less-invasive ROP therapeutic strategies with targets other than VEGF are critically needed to improve the ROP management and the quality of life for a growing number of premature infants.

We propose that drugs targeting adenosine (particularly A_2A_) receptor signaling offer therapeutic advantage by selectively controlling pathological angiogenesis with minimal effect on normal retinal and brain development (for a review see Chen et al. 2017). The validity of A_2A_R signaling as a promising and novel therapeutic target is supported by rapid (minutes) local increase of adenosine level associated with upregulation of enzymes responsible for generating and maintaining adenosine concentration and delayed (~ 24 h) upregulation of A_2A_R in oxygen-induced retinopathy (OIR) model of ROP (Elsherbiny et al. 2013; Lutty and McLeod 2003; Liu et al. 2017), diabetic retina of rat with proliferative retinopathy (Vindeirinho et al. 2013) and other pathological conditions (Chen et al. 2013; Frick et al. 2009; Schingnitz et al. 2010; Linden 2011). Aberrantly increased adenosine-A_2A_R signaling thus represents a *local* “find-me” signal and renders a unique “purinergic chemotaxis” for a *local* resolution to pathological conditions (Chen et al. 2013). Moreover, the variants of human A_2A_R gene are associated with reduced risk of developing diabetic retinopathy in a prospective study (Charles et al. 2011). In support for this proposal, our recent studies with genetic knockout of A_2A_Rs or A_1_Rs suggested that in OIR, the most frequently used model for ocular pathological angiogenesis, genetic inactivation of A_2A_R or A_1_R did not influence normal postnatal development of retinal vasculature, but selectively attenuated pathological angiogenesis (Liu et al. 2010; Zhang et al. 2015), whereas A_1_R activity differentially modulated hyperoxia-induced vaso-obliteration and hypoxia-induced revascularization (Zhang et al. 2015). This proposal is further substantiated by a recent large prospective phase III clinical study “Caffeine Therapy for Apnea of Prematurity” showing severe ROP (as a secondary outcome in a two-year follow-up observation) was less common in infants assigned to chronic caffeine (a non-selective adenosine receptor antagonist) treatment compared with the control (Schmidt et al. 2007), and by pharmacological studies revealing that chronic caffeine treatment selectively attenuates retinal vasculopathy in OIR model (Zhang et al. 2017; Aranda et al. 2016). Thus, A_2A_R antagonism may represent novel and promising pharmacological strategy to control retinal pathological angiogenesis in ROP with distinct advantage over other anti-VEGF antibody strategies, which may be necessary not only for pathological angiogenesis, but also for normal retinal vascularization and brain development (LeBlanc et al. 2013).

However, the effectiveness and selectivity of A_2A_R antagonist-mediated protection against pathological angiogenesis (without affecting normal retinal vascularization) in ROP models have not been tested directly. In this study, the A_2A_R antagonist KW6002, a drug already in clinical phase III trials for Parkinson’s disease treatment with noted safety profile for aging population (Chen et al. 2013), was used to demonstrate the efficacy and selectivity of A_2A_R antagonist control of ROP without affecting normal retinal vascular development. Importantly, contrary to our prediction that A_2A_R antagonists are most effective at the hypoxic phase with aberrantly increased adenosine-A_2A_R signaling, we discovered that the A_2A_R antagonist KW6002 mainly acted at the hyperoxic phase to confer protection against OIR. As ROP being a biphasic disease, targeting the initial vaso-obliteration stage offers therapeutic advantage to preserve developing retinal vascular function and prevent progression to the proliferative phase of ROP. Thus, our identification of the hyperoxic phase as the effective therapeutic window, together with selective and robust protection against pathological (but not physiological) angiogenesis and possible cellular mechanisms (i.e. neuronal cell death and endothelial nitric oxide synthase (eNOS) activity), elevates A_2A_R antagonists as a novel therapeutic strategy for treatment of ROP.

## Methods

### Mouse model of oxygen-induced retinopathy (OIR)

C57BL/6 J mice from the Animal Laboratory of Wenzhou Medical University (Wenzhou, China) were used in this study. The OIR model described previously by Smith and colleagues was adopted (Liu et al. 2010; Smith et al. 1994). Briefly, seven-days old C57BL/6 J mice kept with the foster/nursing mothers were exposed to 75% oxygen for 5 days [from postnatal day 7(P7) to P12 (with P0 being the day for pup delivery)] to induce vaso-obliteration. At P12, the mice were returned to room air (21% oxygen) to induce retinal neovascularizaion, which was maximal at P17. Age-matched mice kept in room air throughout postnatal development (P0-P17) served as “Room-air Controls”. The foster mothers were rotated between the mice exposed to hyperoxia and the mice kept in room air every 8 h.

All procedures with animals in this study were performed in accordance with the Association for Research in Vision and Ophthalmology (ARVO) Statement for the Use of Animals in Ophthalmic and Vision Research and were approved by the Institutional Animal Care and Use Committee of Wenzhou Medical University.

### KW6002 treatment

KW6002 was prepared freshly in 15% DMSO, 15% castrol oil and 70% phosphate buffer saline (PBS), as we described previously (Chen et al. 2001). For each drug treatment, littermates from the same breeding were randomly divided into the drug treatment and control groups. For each treatment condition, at least 2–3 litters were used for the experiment. KW6002 was administered by intraperitoneal injection (i.p.) at dose of 10 mg/kg at different postnatal developmental stages (P7 or P12) and for different period (P7-P17, P7-P12,P12-P17 or P7-P9) every other day or every day. The mice received vehicle in the same volume and with the same intervals served as the control group.

### Fluorescence immunostaining of whole-mount retinas

Fluorescence staining of whole-mounted retinas was performed as previously described (Connor et al. 2009). Mice were euthanized at P12 and P17 and eyes were enucleated and fixed with 4% paraformaldehyde for 1 h. The corneas were removed with scissors along the limbus, and then intact retinas were dissected. Retinas were blocked and permeabilized in PBS containing 0.5% Triton-X-100 overnight at 4 °C. Then retinas were incubated with 10 μg/ml isolectin B4 (Molecular Probes, Life Technologies, Carlsbad, CA, USA)overnight at 4 °C. Retinas were then incubated with anti-glial fibrillary acidic protein (GFAP) mouse monoclonal antibody (1:500, Sigma-Aldrich, St. Louis, MO, USA) for 12 h at 4 °C, followed by incubation with fluorescence-conjugated secondary antibodies (1:500, Invitrogen, Life Technologies, Carlsbad, CA,USA) for 2 h, and then whole-mounted. Retinas were washed with PBS and mounted on microscope slides in mounting medium. Retinas were examined by Laser Scanning Microscope (Zeiss 510; Carl Zeiss). Areas of vaso-obliteration and vitreoretinal neovascular tufts were quantified by using Adobe Photoshop CS 5 software. Eight non-overlapping and randomly selected microscopic fields per retina were imaged by confocal scanning laser microscopy (LSM 710; Carl Zeiss, Oberkochen, Germany) to assess the formation of endothelial tip cells;four non-overlapping and randomly selected microscopic fields in the avascular area per retina were imaged by confocal scanning laser microscopy to assess astrocyte function.

To assess normal postnatal development of retinal angiogenesis, C57BL/6 J mice littermates received vehicle (breeding in room air) were sacrificed and the eyes were harvested at P17, respectively. Whole-mount retinas were stained with isolectin B4. Eight non-overlapping and randomly selected microscopic fields per retina and whole-mounted retina were assessed for morphology and distribution of retinal vessels at P17. Vessels in the three layers (superficial, intermediate and deep) were skeletonized (Kornfield and Newman 2014), and the total vessel length in each microscopic field was calculated using Image-Pro Plus software. Vessel density was quantified as the ratio of the total vessel length to microscopic field.

### Neovascular quantification

Quantification of neovascularization was performed according to the procedure described previously (Liu et al. 2010; Smith et al. 1994). The extent of neo-vascularization was evaluated by counting the number of neovascular nuclei, which were defined as the nuclei of cells extended beyond the inner limiting membrane of the retina into the vitreous body. In this study, eyes of 10 mice from each group were examined and analyzed. For each eye, 20 retinal sections (excluding the optic nerve) were evaluated. All neovascular nuclei were counted under 400× magnifications with hematoxylin and eosin–stained retinal sections by an investigator who was blind to the specific group assignment.

### Semi-quantification of astrocyte in retinal Vaso-obliteration areas

After OIR exposure, mouse retinas were harvested on P17 to assess the astrocyte. GFAP staining of whole-mounted retinas were examined by fluorescence microscopy. The extent of astrocyte persistence in the obliterated zones were scored by the blind observer using the semi-quantitation method on a 1–6 scale as described previously (Dorrell et al. 2010; Liang et al. 2012). As indicated in Fig. 3e, the score of 1–2 indicated retinas with a large number of astrocytes in the vascular obliterated areas which formed a nearly normal astrocytic template. The score of 3–4 indicated retinas with substantial numbers of astrocytes remaining in the vascular obliterated areas (fewer than normal), but failed to form normal astrocytic template. The score of 5–6 indicated there were very few astrocytes in the vascular obliterated zone.

### Terminal deoxynucleotidyl transferase biotin-dUTP nick end labelling (TUNEL) assay

Retinal cell apoptosis was evaluated at P12 or P17. TUNEL staining was performed with the Roche In Situ Cell Death Detection kit (Roche Diagnostic, Basel, Switzerland) following the manufacturer’s instructions. Ten-micrometer–thick cryostat sections with optic nerve head were permeabilized and antigen retrieval was performed by 0.1% sodium citrate buffer solution with 0.5% Triton X-100 for 5 min. After washing 3 times, the sections were incubated in TUNEL reaction solutions for 1 h in 37 °C, then washed and stained for another 5 min using DAPI (1:2000; Beyotime Biotechnology). TUNEL-positive cells were evaluated in three sections crossing the optic nerve per retina under fluorescent microscopy (Zeiss 510; Carl Zeiss).

### Immunohistochemistry

Mouse eyes were dissected and embedded in paraffin at P17 of OIR. For anti–proliferating cell nuclear antigen (PCNA) staining, after being deparaffinized and heated in 10 mM sodium citrate for antigen repairing, 3 retinal paraffin sections were blocked and permeabilized, then incubated with PCNA rabbit polyclonal antibody (1:200; Santa Cruz Biotechnology, SantaCruz, CA, USA). Fluorescence-conjugated secondary Abs (1:500; Thermo Fisher Scientific) were applied to detect positive signals.

For microglial activation by immunostaining with anti-Iba-1 antibody at P12/P17 of OIR, mouse eyes were dissected and embedded in optimum cutting temperature compound. Three cryostat sections with optic nerve head per retina were blocked and permeabilized, then incubated with the anti-Iba-1 rabbit polyclonal antibody (1:100; Wako, Osaka, Japan) overnight at 4 °C. Fluorescence-conjugated second antibodies (1:500; Invitrogen, Life Technologies) were applied for detecting the positive signal.

### Quantitative real-time reverse transcription PCR

The mRNA level of A_1_R, A_2A_R, VEGFA, and eNOS in retina harvested at P12 was done by the quantitative real-time polymerase chain reaction (qPCR) procedure as we have described previously (Zhang et al. 2017) using the following forward and reverse primers: A_2A_R: forward,5’-CCGAATTCCACTCCGGTACA-3′; reverse, 5’-CAGTTGTTCCAGCCCAGCAT-3′; A_1_R: forward, 5’-ATCCCTCTCCGGTACAAGACAGT-3′; reverse, 5’-ACTCAGGTTGTTCCAGCCAAAC-3′; VEGFA: forward, 5’-GAAAGGGTCAAAAACGAAAGC-3′; reverse, 5’-CGCTCTGAACAAGGCTCAC-3′; eNOS: forward, 5’-TGTGACCCTCACCGCTACAA-3′; reverse, 5’-GCACAATCCAGGCCCAATC-3′. GAPDH: forward, 5′- AGGTCGGTGTGAACGGATTTG-3′; reverse, 5’-TGTAGACCATGTAGTTGA GGTCA-3′.

### Statistical analysis

The data were presented as the mean ± standard error (SE). The effect of KW6002 versus vehicle was analyzed by independent Student’s t-test or Mann-Whitney *U* test. The effects of multiple factors were analyzed by two-way ANOVA followed by Bonferroni *post-hoc* test or Kruskal-Wallis test. These statistical analyses were performed with commercial analytical software (SPSS 25.0) with *p* value < 0.05 being considered as statistically significant.

## Results

### Repeated KW6002 treatment did not affect normal postnatal development of retinal vasculature

**Fig. 1:**
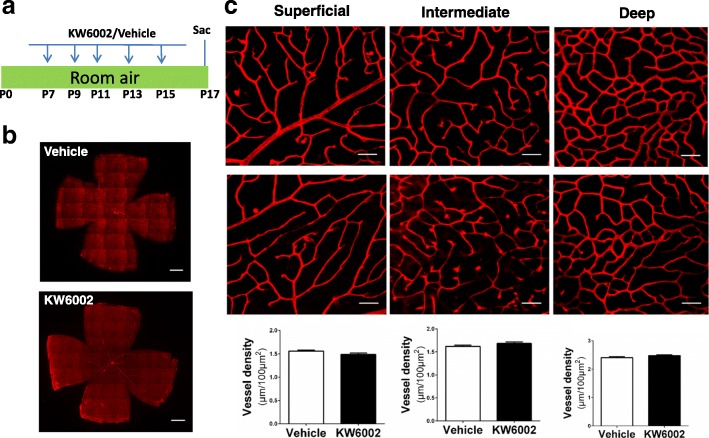
KW6002 treatment did not affect normal postnatal development of retinal vasculature. **a** Schematic of the experimental design: KW6002 was administered by intraperitoneal injection at volume of 10 mg/kg from P7 to P15 every other day. Mice were euthanized at P17.(Sac:Sacrifice). **b** Mouse whole-mount retinas from KW6002- and vehicle-treated mice were harvested and immune-stained with isolectin B4. The retinal vasculature morphologies were indistinguishable between KW6002- or vehicle-treated pups at P17 in room-air. Scale bar: 500 μm. **c** The retinal vasculatures of the superficial, intermediate and deep vascular layers were examined at P17 by isolectin B4 staining of whole-mount retinas. The distributions of three retinal vascular layers were displayed in distinct confocal planes. Vessel density was quantified as the ratio of the total vessel length to microscopic field. The vessel densities of the superficial, intermediate and deep plexuses were indistinguishable between KW6002-treated and vehicle-treated pups. Scale bar: 50 μm

To address whether KW6002 affects normal retinal vasculature during postnatal development, we analyzed development of the retinal vascular networks at P17 of C57BL/6 mice after repeated treatment with KW6002 (from P7 to P17, 10 mg/kg, i.p. every other day, Fig. 1a), by fluorescent staining of whole-mounted retinas under room air conditions (Fig. 1b). After exposure to room air from P0 to P21, the mice displayed normal development of the retinal vasculature, the superficial layer reaching near completion at P12. Both the superficial and the deep vascular layers from the optic disc to the periphery were well developed at P12-P17, and the intermediate layer was almost finished at P21. KW6002 treatment did not affect retinal vasculature in the superficial, intermediate and deep vascular plexus, as revealed by isolectin B4 analysis in whole-mounts retina scanned with laser scanning microscope (Fig. 1c). No avascular areas were detected at P17 in mice treated either with vehicle or KW6002 (Fig. 1b), indicating that the superficial retinal vasculature grew with similar rates and indistinguishable patterns of the vessels distribution between mice treated with vehicle or KW6002. Furthermore, morphology, distribution and density of three retinal vessels layers (superficial, intermediate and deep) were indistinguishable between mice treated with the vehicle and KW6002 when analyzed by confocal scanning laser microscopy at P17 (Fig. 1C). These analyses demonstrated that repeated KW6002 treatment did not affect normal postnatal development of retinal vasculature in mice.

### KW6002 treatment selectively and effectively reduced retinal avascular area and pathological angiogenesis at P17 in OIR

**Fig. 2:**
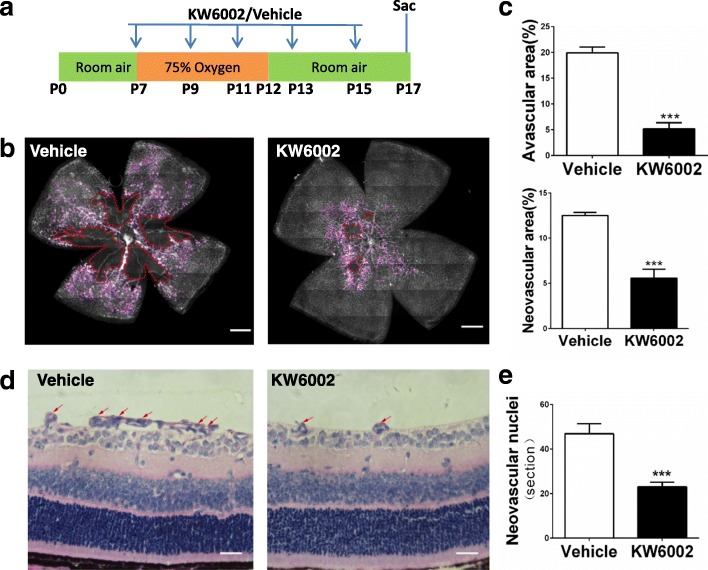
KW6002 treatment attenuated retinal avascular area and pathological angiogenesis at P17 in OIR. **a** Schematic of the experimental design: KW6002 was administered by intraperitoneal injection at volume of 10 mg/kg from P7 to P15 every other day. OIR mice were euthanized at P17. **b** Following the OIR, retinal vasculatures from KW6002- and vehicle-treated mice were visualized by whole-mount isolectin B4 at P17. Avascular areas are indicated by red dotted line. Neovascularization tufts are indicated by purple line. Scale bar: 500 μm. **c** Quantitative analysis of avascular areas by isolectin B4 and the neovascular tufts area showed that KW6002 treatment reduced avascular areas(Student’s t-test) and neovascular tufts area(Mann-Whitney *U* test)compared to the vehicle-treated pups. ***P < 0.001, *n* = 11–13/group. **d** Representative H-E staining images showing the neovascular nuclei numbers (red arrow) on the vitreal side of the inner limiting membrane. Scale bar: 20 μm. **e** Quantitative histochemical analysis showed that KW6002 treatment (P7–17) reduced neovascular nuclei numbers as compared to the vehicle-treated pups. ***P < 0.001, Student’s t-test, *n* = 11–13 /group

We first evaluated the effect of repeated KW6002 treatment for 10 days (from P7 to P17, 10 mg/kg, i.p. every other day, Fig. 2a) on vaso-obliteration and neovascularization by analyzing the avascular area and neovascular area with isolectin B4 staining in whole-mounted retina (Fig. 2b). Repeated treatment with KW6002 largely prevented hypoxia-induced retinopathy. Quantitative analysis demonstrated that KW6002 treatment reduced avascular area by 74.2% (*P* < 0.001) and neovascular area by 55.0% (*P* < 0.001) compared to the vehicle-treated mice (Fig. 2c). Independent and quantitative analysis of neovascular nuclei numbers in cross sections demonstrated that KW6002 treatment for 10 days markedly reduced pathological angiogenesis with reduced neovascular nuclei in retina of OIR (46.85 ± 4.53) compared to vehicle-treated mice (23.03 ± 2.105) (*P* < 0.001, Fig. 2d, e). These studies demonstrated that repeated KW6002 treatment protects against oxygen-induced vaso-obliteration and pathological angiogenesis.

### KW6002-induced protection against OIR was associated with increased astrocyte and endothelial tip cell functions and decreased cellular proliferation and apoptosis at P17

**Fig. 3:**
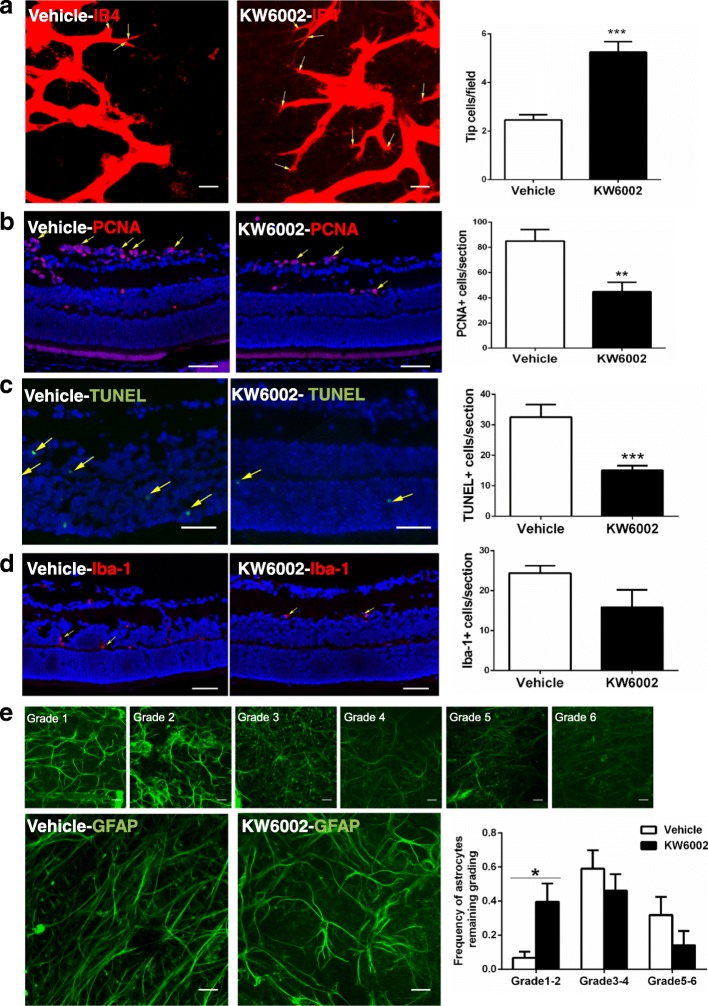
In OIR, the effect of KW6002 treatment on cellular proliferation, tip cell, astrocytes and microglial numbers, and apoptosis in retina at P17. **a** Endothelial tip cells in retina at P17 of OIR were stained with isolectin B4 (red). Scale bar: 20 μm. Quantitative analysis shows that KW6002 treatment increased tip cell number compared to the vehicle-treated pups (***p < 0.001, Student’s t-test, *n* = 11–13/group). **b** Cell proliferation in retina was assessed by immunohistochemistry of PCNA at P17 of OIR. PCNA^+^ cells (yellow arrow) were quantified. (**p < 0.01, Student’s t-test, *n* = 8/group). Scale bar: 50 μm. **c** Apoptotic cells in retina were analyzed by TUNEL (green) staining and individual cells were visualized by DAPI (blue) staining at P17 of OIR. Retinal TUNEL-positive cells (yellow arrow) were quantified and analyzed. KW6002 treatment reduced cellular apoptosis in (**p < 0.01, Mann-Whitney U test, *n* = 8–9/group). Scale bar: 50 μm. **d** Microglial activation in retinas was assessed by immunofluorescence staining of Iba-1 at P17 of OIR. Retinal Iba-1-positive cells (yellow arrow) were quantified and analyzed. (*p* > 0.01, Student’s t-test, *n* = 8/group). Scale bar: 50 μm. **e** Representative images show GFAP-positive cells in the avascular areas by anti-GFAP (green) staining at P17 of OIR. Scale bar: 20 μm. GFAP staining in the avascular area was graded on a scale from 1 to 6 as described in the Methods section. The grades of astrocytes from each treatment group were analyzed at P17 of OIR. KW6002 treatment enhanced GFAP staining (with the reduced grade) (*p < 0.05, Mann-Whitney U test, *n* = 9–12/group), Scale bar: 20 μm

We further investigated the cellular basis underlying the KW6002 effects on pathological angiogenesis by analyzing function of astrocytes and endothelial tip cells in retina of mice treated with KW6002 or vehicle at P17 stage of OIR. Consistent with the previous study (Weidemann et al. 2010), GFAP-positive cells with normal morphology were mainly detected in the peripheral vascular areas while GFAP-positive cells with abnormal morphology were detected in the avascular area of retina by immunofluorescence staining. Using the semi-quantitative method described by Dorrell et al. (Dorrell et al. 2010), we estimated GFAP expression in the avascular area. Analysis showed that OIR markedly reduced GFAP-positive cells, as expected, but KW6002 partially reversed this reduction of astrocyte function in retina compared to the vehicle-treated retina (Fig. 3e). Quantitative analysis revealed that the number of endothelial tip cells in retina was significantly increased by KW6002 treatment compared to the vehicle group (Fig. 3a). Because pathologic angiogenesis is characterized by proliferation of endothelial cells in the hypoxic phase, we analyzed expression of PCNA, a marker for cellular proliferation, at P17 of OIR (Fig. 3b). KW6002 treatment decreased the number of PCNA-positive cells in the retina. TUNEL assay showed that most TUNEL-positive signals were found within the outer nuclear layer, in which mainly photoreceptor cells were distributed (Fig. 3c). Quantitative analysis showed that KW6002 treatment decreased TUNEL-positive signals in retinas compared with vehicle group. Collectively, KW6002 treatment enhanced function of astrocyte and endothelial tip cells and reduced cellular proliferation and apoptosis to confer protection against oxygen-induced pathological angiogenesis in retina at P17 of OIR. Microglial activation in retinas was assessed by Iba-1 immunohistochemistry antibody at P17. OIR increased microglial activation in retina but KW6002 did not have a major effect on microglial activation in retina at P17 (Fig. 3d).

### KW6002 treatment at P7–12 (but not P12–17) was required to confer protection against OIR

**Fig. 4:**
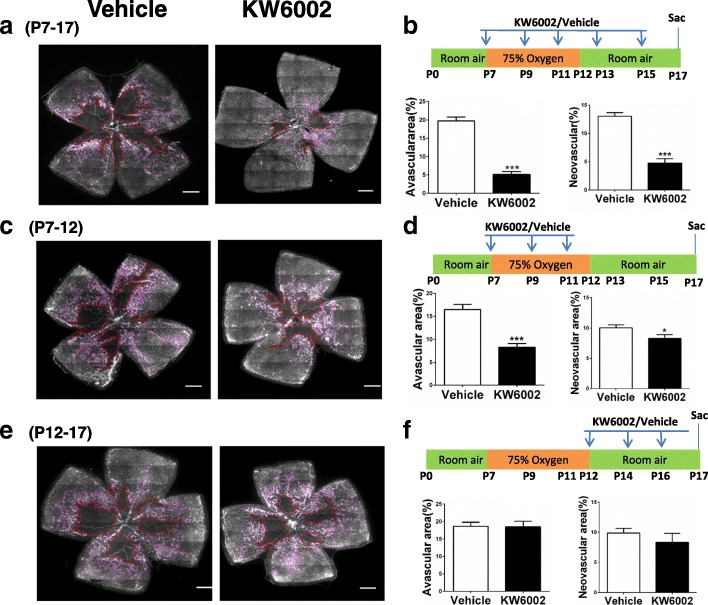
Effective therapeutic windows for KW6002 to confer protection against OIR. **a**, **c**, **e** Pups were treated with KW6002 (10 mg/kg) in different developmental stages (P7–17, P7–12, and P12–17) and retinal vasculatures were analyzed by whole-mount isolectin B4 staining at P17 of OIR. Avascular areas are shown by red dotted line. Neovascularization tufts are indicated by purple line. Scale bar: 500 μm. **b**, **d**, **f** Schematic of the experimental design: KW6002 was administered by intraperitoneal injection at volume of 10 mg/kg at different postnatal developmental stages (P7 or P12) and for different period (P7-P17, P7-P12 or P12-P17) every other day. OIR mice were euthanized at P17. **b**, **d**, **f** Avascular area (%) was quantified as a percentage of the whole retinal surface (*n* = 7–9/group). The neovascularization tufts area (%) was quantified as a percentage of whole retinal area (*n* = 7–9/group). ***p < 0.001, *p < 0.05, Student’s t-test or Mann-Whitney *U* test, *n* = 7–9/group

To define the effective therapeutic window, we treated the mice with KW6002 in three different treatment regimens (i.e. P7-P12, P12-P17 and P7-P17, Fig. 4d, d, f). KW6002 treatment at P7–12 (8.25 ± 0.85%) or 7–17 (5.07 ± 0.82%) was effective in reducing avascular area by 49.9% (*p* < 0.001) and 74.5% (*p* < 0.001), respectively, comparing to the vehicle-treated control (Fig. 4a, b, c, d). In addition, quantitative analysis confirmed that KW6002 treatment at P7–12 (8.29 ± 0.62%) or 7–17 (4.76 ± 0.76%) was effective in neovascularization tufts towards the vitreous body by 17.6% (*p* < 0.05) and 63.44% (*p* < 0.001), respectively, comparing to the vehicle-treated control (Fig. 4a, b, c, d). By contrast, repeated KW6002 treatment at P12–17 was not effective in reducing vaso-obliteration or neovascularization (Fig. 4 e, f). Thus, the administration of KW6002 at P7–12 (i.e. the hyperoxic phase) is required to confer protection against OIR, whereas the injection of KW6002 at P12–17 (i.e. the hypoxic phase) is not sufficient to confer the protection.

### KW6002 treatment reduced hyperoxia-induced retinal vascular regression at P9 and 12 and cellular apoptosis at P12

**Fig. 5:**
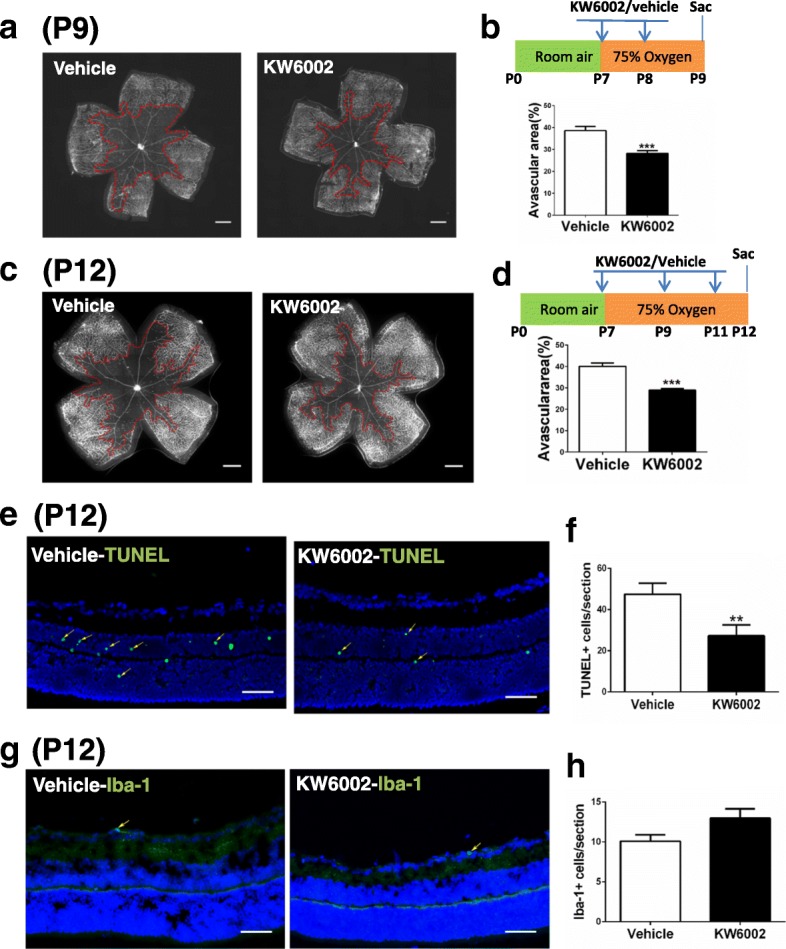
KW6002 treatment reduced hyperoxia-induced retinal vascular regression at P9 and P12 and cellular apoptosis at P12, does not affect the activation of microglia. Whole-mount retinas (**a**, **c**) or retinal cross-section (**e**, **g**) from the vehicle-OIR and KW6002 groups were harvested on P9 (**a**) or P12 (**c**, **e**, **g**) and examined by immunostaining with isolectin B4, or histological analysis by TUNEL or Iba-1. Representative images show the avascular areas (indicated by red dotted line) at P9 (**a**) and P12 (**c**) (Scale bar: 500 μm) and TUNEL^+^ cells (indicated by yellow arrow) (**e**) or Iba-1^+^ cells (indicated by yellow arrow) (**g**) in retinal sections at P12 (Scale bar: 50 μm). Areas of vaso-obliteration (**b**, **d**) and TUNEL^+^ cells (**f**) or Iba-1+ cells (**h**) were quantified and analyzed **P < 0.01, ***P < 0.001, Student’s t-test or Mann-Whitney *U* testn = 8 /group

Next, we further examined the protective effect of KW6002 treatment at the hyperoxic phase by analyzing hyperoxia-induced avascular area at P9 and P12 in whole-mounted retinas with isolectin B4 staining (Fig. 5a,c). Analysis revealed that KW6002 treatment reduced avascular areas in the central retina at P9 and P12. Quantitative analysis of retinal vaso-obliteration demonstrated that KW6002 treatment reduced avascular areas by 27.0% at P9 and 27.5% at P12, respectively, compared to the vehicle-treated mice (Fig. 5d, d).

Consistent with our previously (Zhang et al. 2015) and other studies (Duan et al. 2011; Ludewig et al. 2014; Narayanan et al. 2014), hyperoxia induced TUNEL-positive signals mainly within the inner nuclear layer (mainly neurons such as amacrine cells and bipolar cells) of the avascular area of OIR, in both KW6002- and vehicle-treated groups (Fig. 5e). Quantitative analysis showed that TUNEL-positive cells were lower in mice treated with KW6002 than in mice treated with vehicle at P12 (Fig. 5f). These results confirmed that cellular apoptosis pattern after KW6002 treatment in OIR paralleled with the results of the avascular area, suggesting that KW6002 may reduce avascular areas partly by reducing hyperoxia-induced neuronal apoptosis in retina. We have performed analysis for microglial activation in retinas, and found that KW6002 treatment did not affect microglial activation at P12 (Fig. 5g, h).

### KW6002 treatment reversed hyperoxia-induced reduction of the mRNAs for A_2A_R and eNOS at P12

**Fig. 6:**
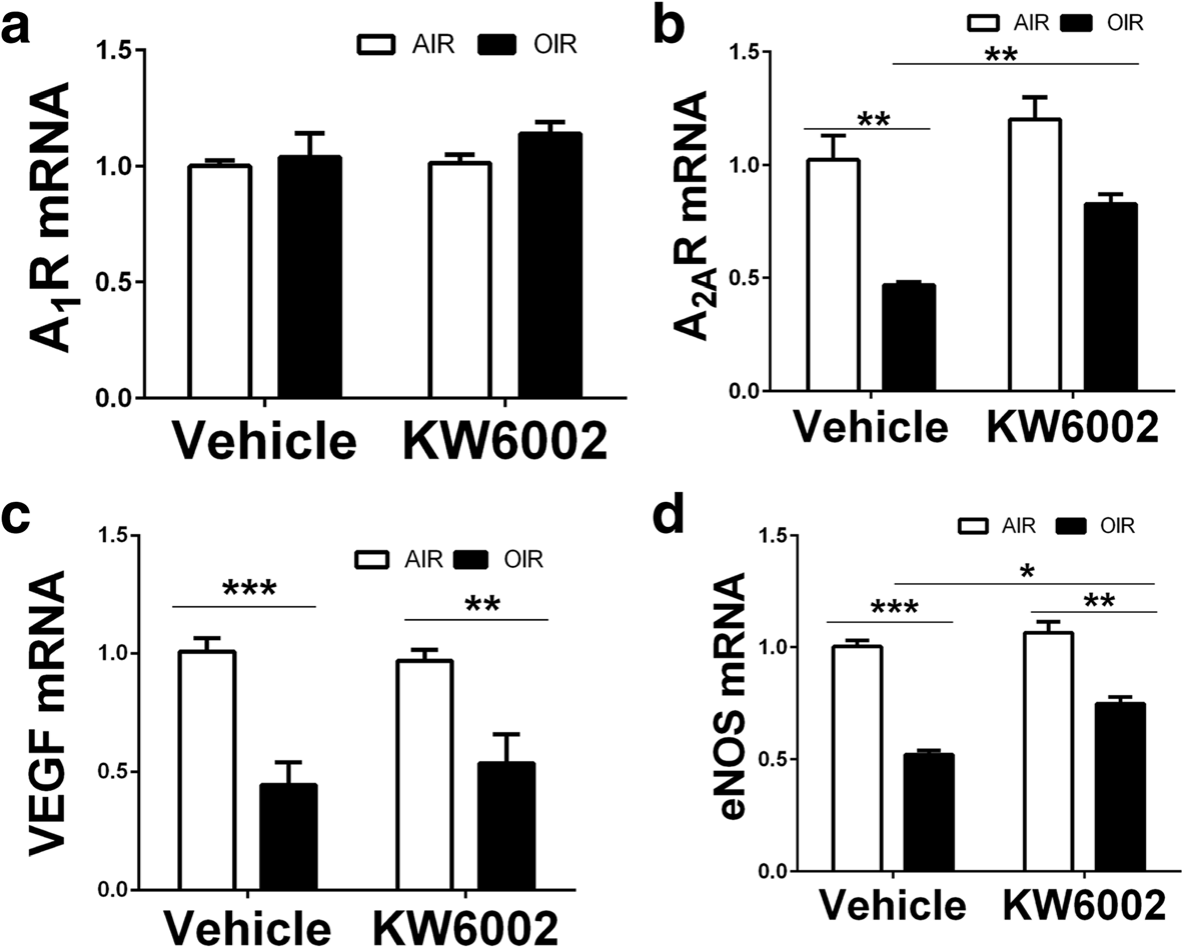
KW6002 treatment reversed hyperoxia-induced reduction of the mRNAs for A_2A_R and eNOS at P12. Retina of OIR and room air was dissected and harvested at P12 in retina after KW6002 or vehicle treatment (P7–12) and A_2A_R and A_1_R mRNAs as well as the mRNAs for VEGF and eNOS were determined by qPCR analysis. Compared to the room air group, A_1_R was affected by neither to hyperoxia or KW6002 treatment (**a**) whereas the mRNAs for A_2A_R, VEGF and eNOS were reduced by hyperoxic exposure. KW6002 treatment reversed the reduction of A_2A_R mRNA and eNOS by hyperoxia (**b**, **c**, **d**). *n* = 6/group *p < 0.05, **p < 0.01, ***p < 0.001, two-way ANOVA followed by Bonferroni post-hoc test or Kruskal-Wallis test *n* = 6/group

We further explored the possible mechanism underlying the A_2A_R-mediated protection at the hyperoxic phase and found that in agreement with the early finding (Taomoto et al. 2000) A_2A_R mRNA was reduced by hyperoxic exposure at P12 (*n* = 6, *p* < 0.01,Fig. 6b). This reduction of the A_2A_R mRNA was reversed by KW6002 treatment. By contrast, A_1_R mRNA in retina of OIR harvested at P12 was affected by neither to hyperoxia or KW6002 treatment (*n* = 6, *p* > 0.05, Fig. 6a). Moreover, as eNOS and VEGF play important role for in the vaso-obliterative phase of ROP (Hartnett et al. 2008), we also examined the expression of eNOS and VEGF at P12 in eye exposed to hyperoxia and after KW6002 treatment (P7–12). Consistent with the previous study (Wang et al. 2013; Abdelsaid et al. 2014), both VEGF mRNA and eNOS mRNA levels were reduced after exposure to hyperoxia compared to the room air condition (*n* = 6, *p* < 0.01) (Fig. 6c, d). Importantly, KW6002 treatment reversed the reduction of eNOS but not VEGF mRNA in eye, suggesting the possible involvement of eNOS in A_2A_R modulation of OIR (independent of VEGF) at the hyperoxia phase.

## Discussion

### A_2A_R antagonists act at the hyperoxic phase to confer its protection against OIR

Current research efforts (such as anti-VEGF and other strategies) have largely focused on the hypoxic phase for proliferative angiogenesis is a key feature of ROP, driven largely by HIF-1α signaling and VEGF pathway. Because hypoxia is associated with huge surge of adenosine signaling as result of increased expression of CD73/CD39 and A_2A_R mRNA and of hypoxia-induced inhibition of ADK and ENT (the enzyme responsible for adenosine degradation), A_2A_R antagonists are expected to act mainly at the hypoxic phase to blunt hypoxia-induced pathological angiogenesis. To our surprise, KW6002 treatment at P12–17 was in fact ineffective, whereas KW6002 treatment at P7–12 was effective in protecting against OIR. KW6002 treatment just at P7–8 was sufficient to confer protection against the damage to the developing retinal vessels (as indicated by reduced avascular area). Lack of protective effect of KW6002 upon administration at P12–17 may be interpreted as follows: direct, acute effect of KW6002 at the hypoxic phase alone is not sufficient and the *combined* effect of KW6002 at both phases is needed to confer its protection against OIR. The reduced pathological angiogenesis by A_2A_R KO at P17 could be attributed to the action at the hyperoxic phase (in addition to the hypoxic phase) as the A_2A_R KO conceives at the early embryonic stage and throughout life. The finding of the required treatment of KW6002 at P7–12 is consistent with the protection against OIR by caffeine observed at P12 (Zhang et al. 2017), and with the finding of the importance of the hyperoxic phase in A_1_R-mediated modulation of OIR (Zhang et al. 2015). Developmental factors may also contribute to the selective effect of A_2A_R antagonists at the hyperoxic phase. Retinal vasculature development at postnatal day 7 might be particularly sensitive to pharmacological interference, since from P7 onward the superficial capillaries start sprouting vertically in the retina to form, firstly, the deep, then the intermediated vascular plexus in the vitreous body of C57BL/6 mice (Smith et al. 1994; Stahl et al. 2010). In addition, KW6002 concentration may be higher at P7-P12 than P12-P17 following the same dose regime because of different metabolism of drugs during postnatal development, as evidenced by caffeine treatment at different postnatal stages producing different steady-state levels of caffeine in human and rodents (Parsons and Neims 1981; Pearlman et al. 1989).

Intriguingly, despite clear vaso-obliteration at the retina center, the hyperoxic phase is associated with lack of “hypoxia” in the retina by in vivo labelling of hypoxic tissues/cells with nitroimidazole EF5 (Smith et al. 2012) and with the reduced expression of ecto-5′ nucleotidase (CD73) and A_2A_R (Lutty and McLeod 2003) and, presumably, adenosine level. Yet, the hyperoxic phase (P7–12 in OIR) is most critical to confer protection against OIR by KW6002, caffeine (Zhang et al. 2017) and A_1_R KO (Zhang et al. 2015). Where did adenosine level come from at the hyperoxic phase? This may be due to the fact that adenosine production is intrinsically linked to energy metabolism and biosynthetic processes (Chen et al. 2013), generating basal levels of adenosine intracellularly and extracellularly through effective bidirectional ENT activity. Consequently, there is always a finite amount of extracellular adenosine that is likely sufficient to activate the A_2A_R (with K_d_ value at 1–10 nM) (Chen et al. 2013). Thus, despite low but nonetheless sufficient concentration of adenosine, KW6002 treatment primarily acts at the early stage of ROP to confer its protection.

The biphasic disease of ROP suggests that hyperoxic damage to developing retinal vasculatures is the primary and root cause of pathological angiogenesis in ROP despite the fact that pathological angiogenesis, the hallmark of ROP pathology, is most evident at the hypoxic phase of ROP (Sapieha et al. 2010). Thus, therapeutic strategies targeting the initial obliteration at the hyperoxia phase can preserve retinal endothelial and neuronal functions and to prevent progression to the proliferative phase of the disease. Our demonstration of the effective therapeutic window (i.e. P7–12) of KW6002 treatment argues that KW6002 treatment is more effective when administered during the early stage of ROP (i.e. when premature infants receive oxygen support) to prevent the devastating latter stage (the hypoxic phase) of the disease. Currently, caffeine treatment for sleep apnea of prematurity is used during the first 10 days after birth in preterm infants subjected to oxygen treatment (Schoen et al. 2014). A_2A_R antagonists and caffeine may be developed as *prophylactic* measures for ROP by targeting the hyperoxic and hypoxic phases to achieve maximal therapeutic benefits.

### A_2A_R antagonists protect against OIR by acting at the distinct mechanisms at the hyperoxic and hypoxic phases

The mechanisms underlying A_2A_R antagonist-induced protection at the hyperoxic phase are not clear. Hyperoxia-induced reactive oxygen species (ROS) can promote apoptosis in ganglia cells and developing endothelial cells, and can inhibit endothelial cells proliferation and migration, leading to vaso-obliteration (Aiello et al. 1994; Alon et al. 1995). Accordingly, attenuation of the hyperoxia-induced avascular area by the A_2A_R antagonist is associated with the reduced neuronal apoptosis in the inner nuclear layer of the retina (i.e. TUNEL-positive cells). This is consistent with the previous finding that A_2A_R antagonists and A_2A_R KO exert protection against OIR (Liu et al. 2010) and seizure susceptibility (Georgiev et al. 1993) in neonates. In keep with this, caffeine treatment during the first 7 days after birth affected neonates sensitivity to ischemic brain injury (Bona et al. 1995), reduced white matter injury by preventing early differentiation of oligodendrocytes (Rivkees and Wendler 2011) and reduced the effects of NMDA on seizure susceptibility in neonates (Georgiev et al. 1993)*.* As the A_2A_R activation can increase Nox2-dependent generation of ROS in some cell types (Ribe et al. 2008; Thakur et al. 2010), A_2A_R antagonists may attenuate cellular apoptosis in the inner ganglion cells by reducing ROS production by hyperoxic exposure. Furthermore, hyperoxic exposure has been shown to impair endothelium function (Garcia-Quintans et al. 2016) with depletion of the eNOS cofactor (6R)-5,6,7,8-tetrahydrobiopterin (BH4) (Edgar et al. 2015). Our observations that KW6002 treatment reversed the reduction of hyperoxia-induced of eNOS mRNA at P12 suggests that A_2A_R antagonists may specifically modulate hyperoxia-induced apoptosis and damage to developing retinal vessels through eNOS-ROS pathway. However, the interplay of the A_2A_R activity and eNOS in OIR are complex because eNOS can have both beneficial (via vasodilation) (Dimmeler et al. 1999) and detrimental (via production of ROS) (Brooks et al. 2001; Edgar et al. 2012) effects on retinal vascular (Edgar et al. 2012) development and because A_2A_R activation can either enhance (Carlstrom et al. 2011)or suppress (Dai et al. 2010; Lin et al. 2007) NOS activity depending on cellular type or local environment (e.g. glutamate). On the other hand, KW6002 reduced avascular areas and promoted revascularization by enhancing the functions of astrocytes and endothelial tip cells at the hypoxic phase at P17. Furthermore, a recent study with endothelial cell-specific A2AR knockout elegantly shows that A2AR control pathological neovascularization in the OIR mice by transcriptional modulation of glycolytic enzymes, via ERK- and Akt-dependent translational activation of HIF-1α protein, to promote glycolysis in endothelial cells and endothelial cell proliferation (Liu et al. 2017).

In this regard, we noted that both A_2A_R antagonist (Fig. 5c, d) and A_1_R KO (Zhang et al. 2015) reduced the oxygen-induced avascular areas at P12. Thus, adenosine acting at A_2A_R and A_1_R corporately controls oxygen-induced damage to developing retinal vasculature at the hyperoxic phase. This is in striking contrast to the fact that A_2A_Rs and A_1_Rs exert opposite control on angiogenesis at the hypoxic phase in OIR, as indicated by reduced avascular areas in A_2A_R-KO mice (Liu et al. 2010) and increased avascular areas in A_1_R-KO mice (Zhang et al. 2015). The different interactions between A_2A_R and A_1_R at the hyperoxia versus hypoxic phases likely reflect fundamentally different mechanisms of adenosine signaling action at these two phases. It would be critical to determine the retinal adenosine level at the hypoxic phase and to dissect out the specific mechanism of the A_1_R and A_2A_R interactions at P12 versus P17.

### Distinct features of A_2A_R antagonists versus anti-VEGF and caffeine therapeutic strategy in ROP

The present study also revealed additional features of A_2A_R antagonists in control of pathological angiogenesis with translational implications for ROP. At first, in contrast to anti-VEGF and other therapeutic strategies, A_2A_R antagonism selectively attenuated retinal pathological vaso-obliteration and neovascularization in OIR, but did not affect postnatal retinal vascularization (with normal morphology, density and distribution of retinal vessels), a feature shared by other adenosine-based manipulations including caffeine treatment (Zhang et al. 2017) and A_2A_R KO (Liu et al. 2010) and A_1_R KO (Zhang et al. 2015). However, the lingering concerns on the possible specific effect of the caffeine on embryonic development (Ma et al. 2014; Back et al. 2006) and possible postnatal development and maturation of cortical GABAergic neurons at the microstructural level after perinatal exposure to the caffeine (Favrais et al. 2014) should be taken into consideration carefully. This treatment confers distinct advantages over the currently testing anti-VEGF antibody treatment in ROP. This selectivity has been attributed to the aberrantly enhanced adenosine signaling (Lutty and McLeod 2003), which amplifies A_2A_R antagonist effect. However, our demonstration of the primary action of A_2A_R antagonists at the hyperoxic phase (with presumably relatively low adenosine signaling) suggests that additional mechanisms may be critical for preferential control of retinal vascularization by A_2A_R signaling at the hyperoxic phase. At second, protective effect of the A_2A_R antagonist against OIR (≈75% avascular area by KW6002 at P17) is stronger/more robust than caffeine treatment (35%) and other experimental manipulations of adenosine signaling, including A_2A_R KO (~ 60%). The robust protection conferred by the A_2A_R antagonist may be attributed to the unique feature of A_2A_R antagonists targeting primarily at the hyperoxic phase. As such, A_2A_R antagonists are more effective at the hyperoxic phase (i.e. the early stage of ROP), whereas anti-VEGF treatment is more effective at the hypoxic phase (i.e. later stage of ROP, such as ROP stage II-III).

## Conclusion

Our identification of the hyperoxic phase as the effective window, together with selective and robust protection against pathological (but not physiological) angiogenesis, provide the preclinical evidence for translating A_2A_R antagonists as a novel therapeutic strategy for ROP treatment.

## Abbreviations

A2AR: Adenosine A2A receptor
eNOS: Endothelial nitric oxide synthase
HIF-1α: Hypoxia-induced factor-1α
OIR: Oxygen-induced retinopathy
ROP: Retinopathy of prematurity
VEGF: Vascular endothelial growth factor
